# Metabolomic Diversity in *Polygonatum kingianum* Across Varieties and Growth Years

**DOI:** 10.3390/molecules29215180

**Published:** 2024-11-01

**Authors:** Liangjun Xiao, Huimei Xu, Tao Wu, Qiufeng Xie, Rouyuan Wen, Le Wang, Baoshun Su, Haizhu Zhang

**Affiliations:** 1 Yunnan Academy of Forestry and Grassland, Kunming 650201, China: xiaoliangjun@yafg.ac.cn (L.X.); wutao@yafg.ac.cn (T.W.); 2 College of Pharmacy, Dali University, Dali 671000, China; xhm19682020@163.com (H.X.); xx184327657@163.com (Q.X.); 15828608051@163.com (R.W.); wl10192391@163.com (L.W.); 3 Linyun Biotechnology Development Co., Ltd., Dali 671000, China

**Keywords:** *Polygonatum kingianum*, varieties, growth years, metabolomics, differential metabolites

## Abstract

*Polygonatum* rhizome is a traditional Chinese medicine of the same origin as food and medicine, and it has high economic value and social benefits. To screen the excellent germplasm resources of *Polygonatum kingianum* (*P. kingianum*) and clarify the nutritional and medicinal value of the rhizome of *P. kingianum*, we used widely targeted metabolomics to analyze the traits and metabolomics of rhizomes of different germplasms of *P. kingianum* from different growth years. The results showed that different germplasms and growth years of *P. kingianum* were rich in different nutritional and medicinal components. Among them, *Polygonatum kingianum* ‘Linyun 1′ rhizome (PWR) was richer in amino acids and derivatives, alkaloids, and phenolic acids, while *Polygonatum kingianum* rhizome (PRR) was richer in flavonoids, organic acids, and phenolic acids. Most of the differential compounds were mainly enriched in PRR when the growth year was one, and PWR had a greater variety and higher content of differential compounds in the third year, which also reflected the advantages of *Polygonatum kingianum* ‘Linyun 1′ (*P. kingianum* ‘Linyun 1′) as an excellent new variety of *P. kingianum*. The Kyoto Encyclopedia of Genes and Genomes (KEGG) metabolic pathway analysis showed that in *P. kingianum* with the same age and different germplasms, the significantly enriched metabolic pathway was more active in biosynthesis in PWR. In the same germplasm of *P. kingianum* from different years, the metabolites involved in PRR were mainly the highest in one-year-old *P. kingianum* (PR-1) or three-year-old *P. kingianum* (PR-3), and the metabolites involved in PWR were mainly the highest in three-year-old *P. kingianum* ‘Linyun 1′ (PW-3). The above results showed that the three-year-old PWR had more advantages based on chemical substances. Therefore, this study provided a new theoretical reference for the development of *P. kingianum* products and the breeding of new varieties.

## 1. Introduction

*Polygonatum kingianum* Coll. et Hemsl (*P. kingianum*) is a perennial herb of the genus *Polygonatum* in the Liliaceae family, and the rhizome is used as medicine or food [1]. It is mainly produced in Guizhou, Guangxi, Yunnan, and other places, and Yunnan is the most important distribution area of *P. kingianum*. As one of the three medicinal *Polygonatum* species, *P. kingianum* is included in the 2020 edition of the Chinese Pharmacopoeia [2]. The rhizome of *P. kingianum* is rich in polysaccharides, saponins, alkaloids, flavonoids and other chemical components [3,4,5]. Additionally, the above components have the effects of enhancing immunity, anti-aging, reducing blood sugar levels, anti-inflammatory and antibacterial [6,7,8]. *P. kingianum* has high medicinal value and broad development prospects in nutrition and health care, health preservation, and the prevention of senile diseases, and so the market demand is expanding. However, due to its slow growth rate, excessive excavation, and environmental degradation, wild *P. kingianum* resources are decreasing and facing depletion [9]. In addition, there is a certain lag in the domestication of wild *P. kingianum*, with problems such as a high incidence of disease and low survival rate [10]. Problems such as mixed varieties of artificially planted *P. kingianum*, unstable quality, and an irrational layout of varieties are prominent, affecting the quality and yield of *P. kingianum* and seriously restricting the stable development of the *P. kingianum* industry [11]. Therefore, studying the key technologies of *P. kingianum*, such as fine variety breeding, high-quality seedling cultivation, standardized germplasm, and precise harvesting, breeding excellent *P. kingianum* germplasm resources suitable for planting, promoting the development and utilization of *P. kingianum* functional products, improving the medicinal value of *P. kingianum*, and even playing a positive role in promoting the development of *P. kingianum* industry, is necessary.

According to research, the synthesis and accumulation of plant secondary metabolites are related to many factors, such as heredity, growth and development stages, geographical location, etc. [12]. The expression and content of active substances in different varieties of the same species are different [13,14]. Similarly, the chemical components of the same variety of plants in different growth years and geographical environments would also be different [15,16]. Variety is one of the key factors determining the yield and quality of medicinal plants [17]. Li et al. [18] found that the accumulation of flavonoids in *P. kingianum* was higher than that in *Polygonatum cyrtonema* Hua (*P. cyrtonema* Hua) and *Polygonatum sibiricum* Red (*P. sibiricum* Red), and the accumulation of phenolic acids in *P. cyrtonema* Hua was significant, while the accumulation of amino acids and derivatives in the three *Polygonatum* species (*P. kingianum*, *P. cyrtonema* Hua, and *P. sibiricum* Red) was significant and the contents were quite different. Additionally, the growth year can significantly affect the accumulation of active ingredients in tuber medicinal materials [19]. A related study showed that the accumulation of polysaccharides in *P. kingianum* was greatly affected by the growth age, and the polysaccharide content of 4-year-old *P. kingianum* was the highest [20]. In the growth process of *P. kingianum*, there was a material transfer between different growth years, and the dry weight and quality of plants from each year were inconsistent. If confused, it would cause a decrease in efficacy [21]. In conclusion, the effects of the cultivar and growth year on the accumulation of plant secondary metabolites are complex. Therefore, as a valuable traditional Chinese medicine, studying the effects of varieties and growth years on the contents of secondary metabolites in the *P. Kingianum* rhizome is necessary.

In recent years, research on *P. kingianum* mainly focused on polysaccharides and saponins [22,23]. However, research on other medicinal components in *P. kingianum* is limited, and the differences in small-molecule compounds in vivo could not be analyzed in detail. In addition, there were few studies that analyzed the overall metabolites of different varieties of *P. kingianum* in different growth years, which was not conducive to the effective utilization of new varieties of *P. kingianum*. Metabolomics can be used to comprehensively analyze the changes in metabolite contents and analyze the changes in differential metabolites between different samples due to different planting conditions, harvesting times, processing methods, etc. [24], thus assisting in the selection of dominant varieties or the best processing methods. Widely targeted metabolomics is characterized by high throughput, ultra-sensitivity, and broad metabolite coverage, allowing the simultaneous quantification of hundreds of known metabolites and nearly 1000 unknown metabolites [25]. Nowadays, this technology has been recognized. In addition to research on traditional economic crops [26], it has also been applied to quality control and quality evaluation, germplasm identification, plant classification, kinship assessment, active ingredient metabolic pathways, and related regulatory mechanisms of traditional Chinese medicine [27,28,29].

*Polygonatum kingianum* ‘Linyun 1′ (white flower, *P. kingianum* ‘Linyun 1′) is a new variety of *P. kingianum* that was identified by the Forest Variety Approval Committee of Yunnan Province, China, in 2019, and its polysaccharide content was 15.2% [30], which was higher than *P. kingianum* planted in other places in Yunnan. It has been reported that the overall transpiration rate and total chlorophyll content of *P. kingianum* ‘Linyun 1′ were higher than those of *P. kingianum* with red flowers [31]. To explore the reasons for the high yield of PWR and enrich the germplasm resources of *P. kingianum*, we conducted an ultra-performance liquid chromatography mass spectrometry (UPLC-MS/MS) metabonomic analysis of different varieties of *P. kingianum* from different growth years, explored the differences in metabolic components between traditional *P. kingianum* and its new varieties, and clarified the accumulation of metabolites in *P. kingianum* from different growth years in order to provide a reference for the selection and comprehensive utilization of quality *P. kingianum* germplasm resources.

## 2. Results and Discussion

### 2.1. Phenotypic Differences Among Different Germplasms of P. kingianum

**Figure 1:**
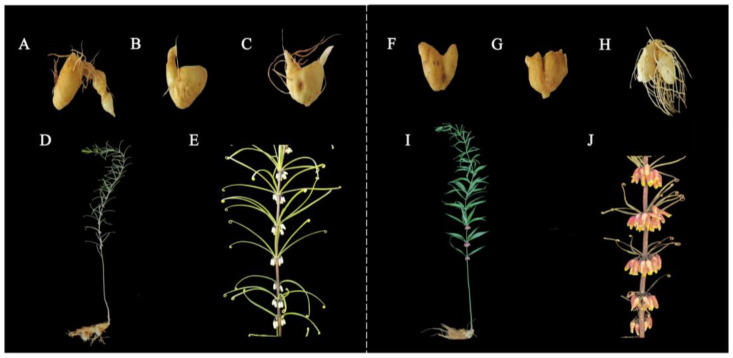
Phenotypic characteristics of different *P. kingianum* germplasms from different growth years. (**A–C**) One-to-three-year-old PW (*Polygonatum kingianum* ‘Linyun 1′); (**D**) plants of PW; (**E**) flowers of PW; (**F–H**) one-to-three-year-old PR (*Polygonatum kingianum*); (**I**) plants of PR; and (**J**) flowers of PR.

PWR and PRR were the rhizomes of two *P. kingianum* germplasms used in this study. Under the same growth conditions, we found that *kingianum* ‘Linyun 1′ had a white perianth, slightly smaller tubers, and fewer fibrous roots; *P. kingianum* had a red perianth, slightly larger tubers, and more fibrous roots (Figure 1). In addition, we used a random sampling method to observe the plant height, plot size, and seedling emergence of 600 plants of both *P. kingianum* germplasms from 32,000 plants. As can be seen in the table, the average plant height of 1~4-year-old traditional *P. kingianum* plants was higher than that of 1~4-year-old *P. kingianum* ‘Linyun 1′ plants, but the average ground diameter and the number of plants per cluster of *P. kingianum* ‘Linyun 1′ were higher than those of traditional *P. kingianum* (Table 1). It is worth noting that the average yield per plant and average yield per mu of four-year-old *P. kingianum* rhizome were more prominent in PWR (Table 2). The above results showed that compared with the traditional *P. kingianum*, *P. kingianum* ‘Linyun 1′ was shorter, more resistant, had more buds, had fewer fibrous roots, and had a higher yield. Based on these results, an extensive targeted metabolomic analysis was performed to characterize the metabolic differences between the two germplasms.

### 2.2. Analysis of the Overall Composition of Metabolites

**Figure 2:**
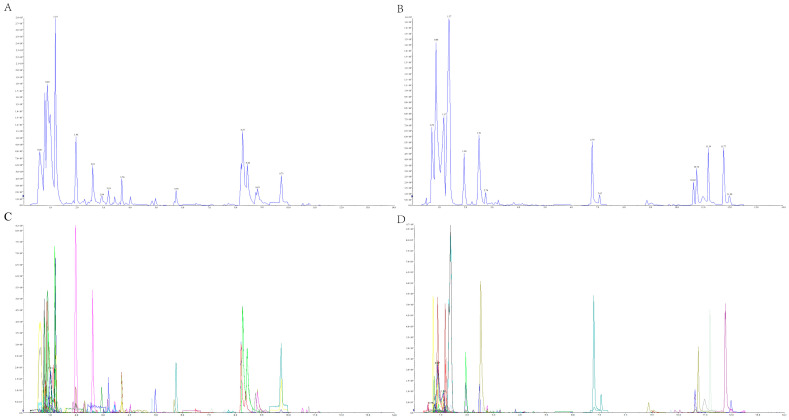
TIC of quality control (QC) samples and multi-peak chromatograms of metabolites using MRM. (A) positive TIC chromatogram of QC samples; (B) negative TIC chromatogram of QC samples; (C) Positive ion current multi-peak chromatograms of metabolites in quality control samples using MRM; (D)negative ion current multi-peak chromatograms of metabolites in quality control samples using MRM.

**Figure 3:**
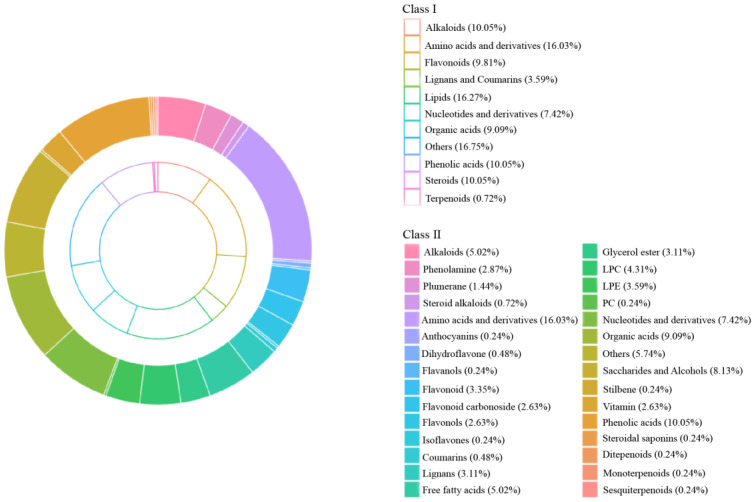
Distribution of metabolites in PWR and PRR from different growth years.

To better understand the metabolites in different germplasms of *P. kingianum* from different growth years, the primary and secondary metabolites were identified by UPLC-MS/MS. Figure 2A,B illustrates the total ion current (TIC) maps of the quality control sample. The TIC maps showed the total intensity of all ions in the mass spectrum over time. Figure 2C,D shows the multi-peak chromatograms of metabolites in MRM mode. In the multi-peak chromatograms, peaks with different colors represented the detected metabolites. Through the metabolite database, the qualitative and quantitative analyses were carried out according to the metabolite ion information for the rhizome of *P. kingianum*. A total of 418 metabolites were identified, which were divided into 11 categories, including 68 lipids, 67 amino acids and derivatives, 42 phenolic acids, 42 alkaloids, 41 flavonoids, 38 organic acids, 31 nucleotides and derivatives, 15 lignans and coumarins, 3 terpenoids, 1 steroid, and 70 other metabolites. The proportions of various types of chemical components are shown in Figure 3. Detailed information on metabolites is shown in Table S1. A total of 409, 412, 407, 412, 408, and 404 metabolites were detected in PW-1, PW-2, PW-3, PR-1, PR-2, and PR-3, respectively. It was found that the total amount and types of metabolites detected in PWR were higher than those in PRR, among which amino acids and derivatives, phenolic acids, alkaloids, flavonoids, and lipids were the main metabolites. This study provided a new research idea for the different analyses of different germplasms of *P. kingianum* from different growth years and provides a certain reference for the evaluation and comprehensive utilization of germplasm resources of *P. kingianum* ‘Linyun 1′.

### 2.3. Multivariate Analysis Revealed the Differences Between the Metabolic Profiles

**Figure 4:**
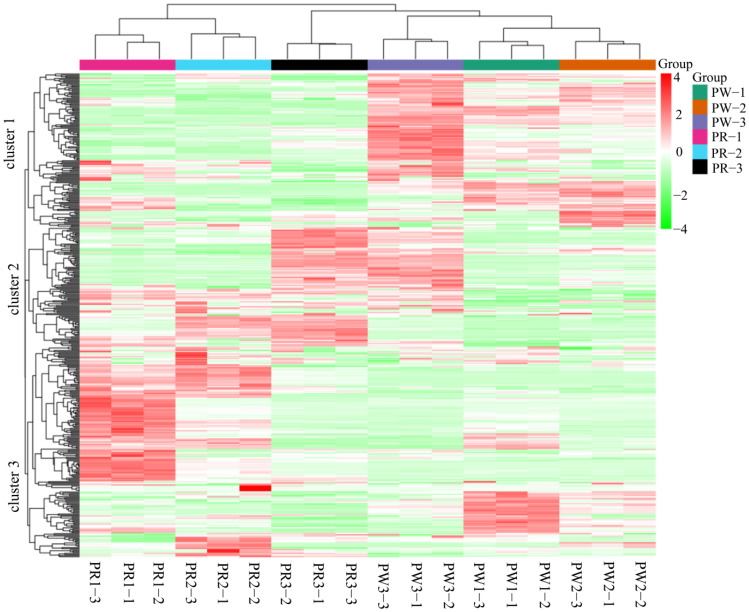
Hierarchical clustering analysis (HCA) and correlation analysis between PWR and PRR samples from different growth years.

**Figure 5:**
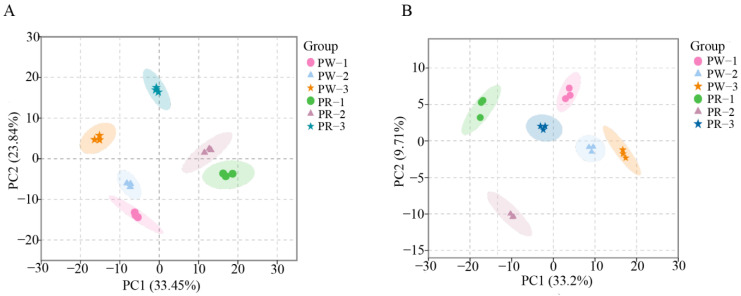
Principal component analysis (PCA) and orthogonal partial least squares discriminant analysis (OPLS-DA) between PWR and PRR samples from different growth years. (**A**) PCA diagram and (**B**) OPLS-DA diagram.

The samples were analyzed by HCA, PCA, and OPLS-DA to discriminate the magnitudes of the variation between and within groups of samples from the PWR and PRR germplasms. The results of HCA showed that there were significant differences between the samples from each group, and all the metabolites with significant differences were divided into three categories (Figure 4). The metabolites of cluster 1 accumulated the most in PW-3 and PW-2, the metabolites of cluster 2 accumulated the most in PW-3 and PR-3, and the metabolites of cluster 3 accumulated the most in PR-2 and PR-1. PCA revealed a trend of separation among the two groups of samples (PWR and PRR), showing a trend of separation on PC1, and the cumulative contribution of PC1 and PC2 is 57.29% (Figure 5A). The OPLS-DA model compared the metabolite contents in PWR and PRR in pairs to evaluate the differences. In the OPLS-DA model of different *P. kingianum* germplasms, all groups were within the confidence interval, the model construction was effective (Table S2), and the prediction accuracy was high, and so it could be used to screen differential metabolites (Figure 5B).

### 2.4. Screening and Analysis of Differential Metabolites in Different Germplasms of P. kingianum from Different Growth Years

**Figure 6:**
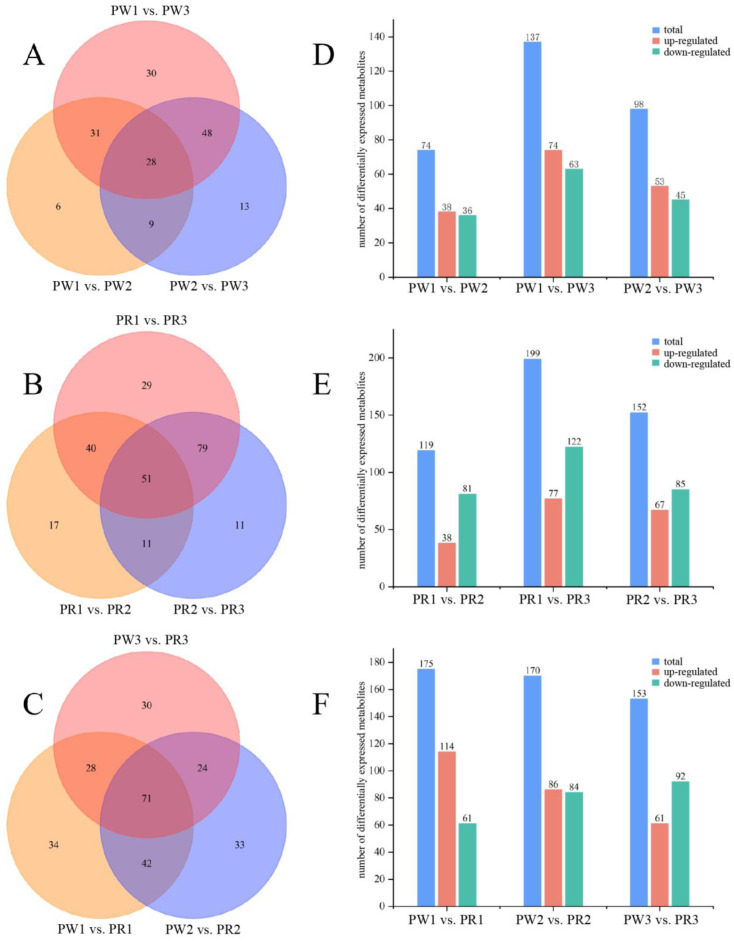
Screening results for differential metabolites in the rhizome of *P. kingianum* between different germplasms and different growth years. (**A**–**C**) Venn diagrams of differential metabolites/(**D**–**F**) The regulation mode of differential metabolites in different comparison groups. (**A**,**D**) One-to-three-year-old PWR; (**B**,**E**) one-to-three-year-old PRR; and (**C**,**F**) PW-1 vs. PR-1, PW-2 vs. PR-2, and PW-3 vs. PR-3.

To further understand the metabolic differences between PWR and PRR in three different growth years, we screened differential metabolites according to FC ≥ 2 or ≤0.5 and VIP ≥ 1. By comparing the screening results of differential metabolites, it was found that the metabolites of different germplasms of *P. kingianum* in the same growth years were significantly different (Figure 6F). When the growth year was one, there were 175 significantly different metabolites between the two varieties of *P. kingianum*, including 114 up-regulated metabolites and 61 down-regulated metabolites. The differential metabolites were mainly flavonoids and lipids. Similarly, when the growth year was two, there were 170 significantly different metabolites in the two varieties, including 86 up-regulated metabolites and 84 down-regulated metabolites. The differential metabolites were mainly flavonoids and amino acids and derivatives. When the growth year was three, there were 153 significantly different metabolites between PWR and PRR, including 61 up-regulated metabolites and 92 down-regulated metabolites. The differential metabolites were mainly flavonoids and amino acids and derivatives. These results indicated that the metabolites of the two *P. kingianum* germplasms are very different, and flavonoids, amino acids and derivatives, lipids, and other metabolites might be the main components that caused the difference between the two *P. kingianum* germplasms. The number of up-regulated metabolites during the growth of one-year-old *P. kingianum* was higher than that of down-regulated metabolites, and most of the up-regulated metabolites were flavonoids, which indicated that the content of most flavonoids in PRR was higher than that in PWR at one year old (Table 3). It was worth noting that when the growth period of *P. kingianum* reached the third year, the number of down-regulated metabolites was higher than the number of up-regulated metabolites, indicating that most of the lipid content in PWR was higher than that in PRR at the age of 3 years.

In addition, we compared the differential metabolites between different growth years in the same germplasm. A total of 212 differential metabolites were screened in PWR (Figure 6D). A total of 137 differential metabolites (74 up-regulated and 63 down-regulated) were identified from the comparison of the PW-1 and PW-3 groups. The significantly up-regulated differential metabolites were mainly lipids, and the significantly down-regulated differential metabolites were mainly alkaloids. Similarly, 98 differential metabolites (53 up-regulated and 45 down-regulated) were identified from the comparison of the PW-2 and PW-3 groups. The significantly up-regulated differential metabolites were mainly lipids and amino acids and derivatives, and the significantly down-regulated differential metabolites were mainly alkaloids. PW-1 and PW-2 groups had the least number of differential metabolites, a total of 74 (38 significantly up-regulated and 36 significantly down-regulated), and the differential metabolites were mainly phenolic acids. Compared with PW-1 and PW-2, the number of up-regulated metabolites in PW-3 was higher than that of down-regulated metabolites, and most of the up-regulated metabolites were lipids. This showed that with the increase in growth years, the lipid content of PWR gradually accumulated and increased.

Similarly, a total of 215 differential metabolites were screened in PRR (Figure 6E). A total of 119 differential metabolites (38 up-regulated and 81 down-regulated) were screened from the PR-1 and PR-2 groups, and most of the differential metabolites were lipids and phenolic acids. In the PR-1 and PR-3 groups, there were 199 differential metabolites, of which 77 were significantly up-regulated and 122 were significantly down-regulated. In PR-2 and PR-3 groups, there were 152 differential metabolites, of which 67 were up-regulated and 85 were significantly down-regulated, most of which were organic acids and flavonoids. Compared with PR-1 and PR-2, the number of down-regulated metabolites in PR-3 was higher than that of up-regulated metabolites, and most of the down-regulated metabolites were flavonoids. This indicated that most of the flavonoids in PR-3 were present at lower levels than those in PR-1 and PR-2, and the accumulation of flavonoid metabolites decreased with age.

The results of the Venn diagram showed that twenty-eight common differential metabolites in eight categories were screened out in the comparison of PWR groups from different growth years (Figure 6A), including seven alkaloids, three phenolic acids, three flavonoids, two nucleotides and derivatives, one amino acid and derivative, one organic acid, one lipid, and ten other categories (Table S3), which could be used as markers to distinguish PWR from 1~3 years old. Nine kinds of fifty-one common differential metabolites were screened from PRR comparison groups from different growth years (Figure 6B), including twelve phenolic acids, twelve flavonoids, seven alkaloids, two nucleotides and their derivatives, three amino acids and derivatives, five organic acids, 1 coumarin and lignan, one terpenoid, and eight other categories (Table S4), which could be used as markers to distinguish 1~3-year-old PRR. These common differential metabolites with significant differences in contents in *P. kingianum* from different growth years (Figure S1) might be one of the factors that cause the differences in the efficacy and quality of *P. kingianum* in the three age groups.

### 2.5. Analysis of the Contents of Differential Metabolites

**Figure 7:**
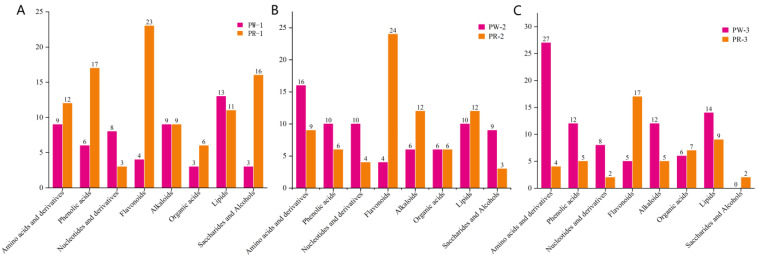
Relative amounts of the highest differential metabolites in different germplasms *P. kingianum*. (**A**–**C**) Relative amounts of the highest differential metabolites in the three comparison groups. Red and orange colors represent PWR and PRR, respectively.

By analyzing the contents of differential metabolites in different germplasms of *P. kingianum* rhizome from different growth years, it was found that some differential metabolites showed regular changes with the increase in growth years, as shown in Figure 6 and Figure 7. Comparing the relative contents of major differential metabolites between the two germplasms, we found that the relative contents of 13 lipids (accounting for 54.17% of the total differential lipid metabolites) in one-year-old PWR were higher (Figure 6D). The relative contents of 16 phenolic acids (69.57%), 23 flavonoids (70.83%), 6 organic acids (66.67%), 12 amino acids and derivatives (57.14%), and 16 saccharides and alcohols (84.21%) in PRR were significantly higher than those in PWR. The relative contents of 16 amino acids and derivatives (64.00%), 10 phenolic acids (62.50%), and 9 saccharides and alcohols (75.00%) were higher in 2-year-old PWR. The relative contents of 24 kinds of flavonoids (85.71%), 12 kinds of alkaloids (66.67%), and 12 kinds of lipids (54.55%) in PRR were significantly higher than those in PWR (Figure 6E). The relative contents of 29 amino acids and derivatives (93.55%), 12 phenolic acids (70.59%), 12 alkaloids (70.59%), and 12 lipids (52.17%) were higher in three-year-old PWR. The relative contents of seventeen flavonoids (77.27%) and seven organic acids (53.85%) in PRR were significantly higher than those in PWR (Figure 6F). The results showed that one-year-old PWR was rich in lipids and nucleotides and derivatives, and PRR was rich in phenolic acids, flavonoids, amino acids and derivatives, organic acids, and saccharides and alcohols. Two-year-old PWR was rich in amino acids and derivatives, phenolic acids, nucleotides and derivatives, and saccharides and alcohols, and PRR was rich in flavonoids, alkaloids, and lipids. Three-year-old PWR was rich in amino acids and derivatives, phenolic acids, alkaloids, nucleotides and derivatives, and lipids, and PRR was rich in flavonoids and organic acids. The above results showed that most of the differential compounds were mainly enriched in PRR when the growth year was one. In the third year, the PWR had more variety and higher contents of differential compounds, and the expression of several metabolites was significantly higher than that in PRR, which indicated that more metabolites had accumulated in PWR relative to PRR. However, whether it was superior to PRR in pharmacodynamic activity and whether it had in vivo pharmacodynamic convergence due to large differences in chemical composition caused by varieties needs further pharmacological verification.

In recent years, there have been a lot of studies on the content and composition of polysaccharides in single *P. sibiricum*, but there were few studies on flavonoids, amino acids, and phenolic acids [20]. These substances are also important medicinal components of *P. sibiricum*. It has been reported that amino acids in plants are the main components for treating diseases or nourishing nutrition [32,33], and they also play an important role in maintaining the balance of plant metabolism [34]. Polyphenols can reduce the increased blood glucose levels in mice induced by streptozotocin and have a hypoglycemic effect [35]. Lipids are one of the most important nutrients in the human body, and they can provide the body with the energy and fatty acids it needs [36]. Among them, choline glycerophosphate GPC is a water-soluble phospholipid metabolite that has many effects, such as lipid-lowering and brain-strengthening effects [37]. In this study, compared with PRR, PWR contained more abundant phenolic acids, lipids, and amino acids and derivatives. Therefore, PWR had a better medicinal value of phenolic acids and lipids, and a higher nutritional value of amino acids. It was speculated that PWR had a broader prospect in the development of pharmaceutical and health products. As an important secondary metabolite in plants, the accumulation of flavonoids is conducive to the improvement of antioxidant, anti-inflammatory, and anti-tumor effects [38]. Organic acids are a class of organic compounds with carboxyl groups. Organic acids in plants play very important roles, such as carbon sources, enhancing plant resistance, affecting amino acid synthesis, bacteriostasis, etc. [39,40]. PRR was richer in flavonoids and organic acids and was presumed to have greater medicinal value in terms of flavonoids and organic acids than PWR. In summary, the two *P. kingianum* germplasms had different nutritional and medicinal components, which could provide a reference for the rational development of medicinal metabolites of medicinal plants.

Our study further found that some metabolites were identified only in one germplasm. Among them, N-(3-indolylacetyl)-L-alanine, L-isoleucyl-L-aspartate, and 6-O-caffeoylarbutin were only detected in PWR, and the relative contents were higher at PW-3. Matairesinol 4,4′-di-O-glucoside*, D-(-)-arabinose, and (25S)-kingianoside A were also identified only in PWR. Limocitrin-7-O-glucoside, chrysoeriol-6,8-di-C-glucoside, neobaicalein, and coselanthine were unique metabolites in PRR. These significant metabolites provided a new research idea for the discrepancy analyses of different germplasms of *P. kingianum* and provided a reference for the excavation and breeding of high-quality germplasms of *P. kingianum*. Studies had reported that 6-O-caffeoylarbutin has a certain hypoglycemic effect and a certain protective effect on the liver, kidney, and pancreas of type I diabetic mice [41]. It was speculated that the unique substances contained in different germplasm of *P. kingianum* could be used and studied in a targeted manner, and the unique metabolites could be used for identification between species.

**Figure 8:**
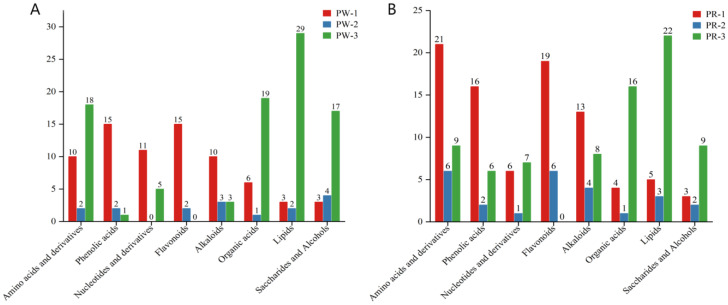
Relative amounts of the highest differential metabolites in *P. kingianum* from different growth years. (**A**,**B**) Relative amounts of the highest differential metabolites in the two comparison groups. Red, blue, and green represent 1 year old, 2 years old, and 3 years old, respectively.

Then, the relative contents of the main differential metabolites in the three age groups (PW-1, PW-2, and PW-3) of PWR were compared. It was found that the relative contents of 15 flavonoids (88.24%), 15 phenolic acids (83.33%), and 10 alkaloids (62.50%) in PW-1 were the highest. The relative contents of 18 amino acids and derivatives (60.00%), 19 organic acids (73.08%), 17 saccharides and alcohols (70.83%), and 29 lipids (85.29%) were the highest in the PW-3 (Figure 8A). Therefore, PW-1 was richer in flavonoids, phenolic acids, and alkaloids than other age groups; PW-3 was richer in amino acids and derivatives, organic acids, saccharides and alcohols, and lipids than the other age groups. Similarly, the relative contents of the main differential metabolites in the three age groups (PR-1, PR-2, and PR-3) of PRR were compared. It was found that the relative contents of 21 amino acids and derivatives (58.33%), 19 flavonoids (76.00%), 16 phenolic acids (66.67%), and 13 alkaloids (52.00%) in PR-1 were the highest. The relative contents of 22 lipids (73.33%), 16 organic acids (76.19%), and 9 saccharides and alcohols (64.29%) were the highest in the PR-3 (Figure 8B). Therefore, the PR-1 was richer in amino acids and derivatives, flavonoids, phenolic acids, and alkaloids than other age groups; the PR-3 was richer in organic acids, saccharides and alcohols, and lipids than other age groups.

Metabolites such as lipids, organic acids, phenolics, and steroids are not exclusively derived from plants. Microorganisms are a promising source of an enormous number of natural products. For example, *Cunninghamella blakesleeana* can produce fatty acids such as palmitic acid, oleic acid, and stearic acid [42]. *Cunninghamella elegans* produces 3-hydroxytyrosol and the QSMs 2-phenylethanol and tyrosol to regulate biofilm growth [43]. 2-Phenylethanol is an endogenous molecule that participates in the biofilm growth of *Cunninghamella echinulata* [44]. Aspergillus sp. and several other yeasts are employed for the production of citric acid. *E. coli*, *Klebsiella oxytoca* and *Clostridium thermocellum* are involved in fermentative production of ethanol using different carbon sources [45]. The results of this study provided a basis for the development and utilization of *P. kingianum* from different age groups. Subsequent research can investigate the relationship between *P. kingianum* and soil microbial communities, and further explore the impact of microorganisms on the accumulation of *P. kingianum* components.

### 2.6. KEGG Functional Annotation and Enrichment Analysis of Differential Metabolites

**Figure 9:**
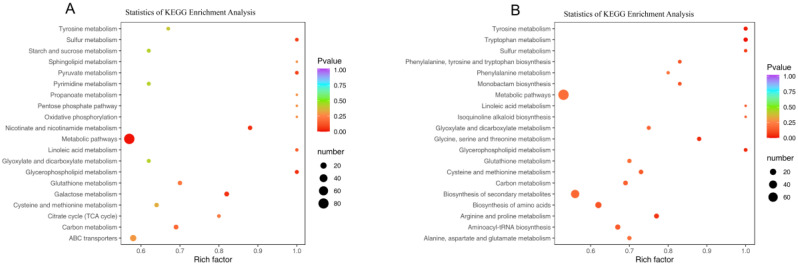
KEGG pathway enrichment analysis of differentially accumulated metabolites (DAMs) in the rhizome of *P. kingianum* between different germplasm and different growth years. (**A**) PW-1 vs. PW-2 vs. PW-3; (**B**) PR-1 vs. PR-2 vs. PR-3.

**Figure 10:**
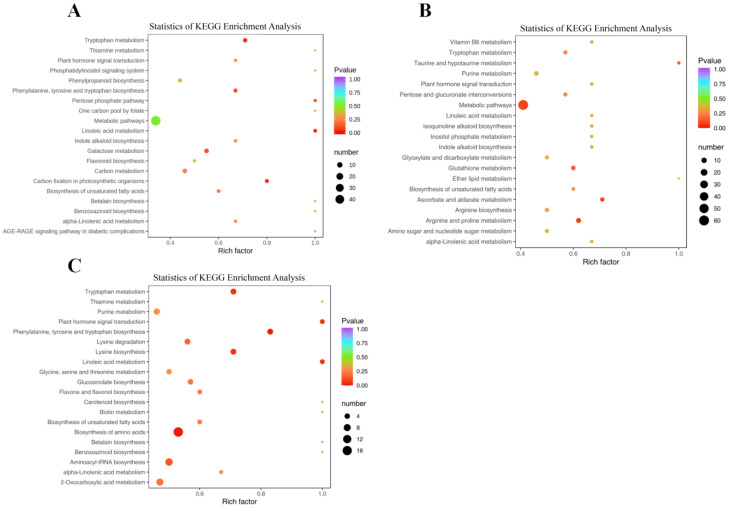
KEGG pathway enrichment analysis of differentially accumulated metabolites (DAMs) in the rhizome of *P. kingianum* between different germplasms and different growth years. (**A**) PW-1 vs. PR-1; (**B**) PW-2 vs. PR-2; and (**C**) PW-3 vs. PR-3.

The pathway enrichment analysis of differential compounds was carried out by the KEGG database. The results showed that the differential metabolites of PW-1 and PR-1 groups were mainly enriched in 69 metabolic pathways, and the significantly enriched metabolic pathways included the biosynthesis of phenylalanine, tyrosine, and tryptophan; linoleic acid metabolism; carbon fixation of photosynthetic organisms; and tryptophan metabolism (Figure 9A). Among them, there were five differential compounds involved in carbon fixation in photosynthetic organisms, all of which are accumulated in PR-1, such as L-aspartic Acid, L-(-)-malic acid, etc. There were seven different compounds involved in tryptophan metabolism, and six metabolites accumulated more in PW-1 than PR-1, which were methoxyindoleacetic acid, N-acetyl-5-hydroxytryptamine, etc. The results showed that 1-year-old PWR was more active in biosynthesis in tryptophan metabolism, and metabolites involved in carbon fixation in photosynthetic organisms were expressed at higher levels in PRR. When *p* < 0.05, there was no significant metabolic pathway for the differential metabolites of PW-2 and PR-2 (Figure 9B). KEGG analysis of PW-3 and PR-3 groups showed that the differential metabolites were mainly enriched in 63 metabolic pathways (Figure 10C), and the significantly enriched metabolic pathways included the biosynthesis of amino acids; phenylalanine, tyrosine and tryptophan biosynthesis; linoleic acid metabolism; and plant hormone signal transduction. There were three differential metabolites involved in plant hormone signal transduction, and jasmonic acid and N-[(-)-jasmonoyl]-(L)-isoleucine (JA-L-Ile) accumulated more in PW-3. There were 80 different compounds involved in the biosynthesis of amino acids, among which 45 metabolites accumulated more in PW-3, mainly L-threonine, L-isoleucine*, and other compounds. Compared with PRR, three-year-old PWR was more active in the biosynthesis of the above metabolic pathways, and its metabolite expression level was higher. Linoleic acid metabolism and phenylalanine, tyrosine, and tryptophan biosynthesis were the pathways in which the differential metabolites were jointly significantly enriched in the two comparator groups (PW-1 vs. PR-1 and PW-1 vs. PR-1). There were three differential compounds involved in linoleic acid metabolism, and two of these metabolites accumulated more in PR-3 than PW-3, namely γ-linolenic acid* and linoleic acid. There were six differential compounds involved in phenylalanine, tyrosine and tryptophan biosynthesis, and four of these metabolites accumulated more in PW-3 than in PR-3, namely indole, L-phenylalanine, L-tyrosine, and L-tryptophan.

It has been reported that linoleic acid is an essential fatty acid for the human body, which could be converted into γ-linolenic acid in the body and then extended to arachidonic acid. Under the action of a series of enzymes, arachidonic acid could be used to synthesize prostaglandins, thromboxane, and leukotrienes [46]. These compounds have strong physiological activities and are related to various pathological processes, such as inflammation, allergic reactions, and cardiovascular diseases. In this study, it was found that the differential metabolites (γ-linolenic acid* and linoleic acid) related to linoleic acid metabolism were highly expressed in PR-3. Phenylalanine, tryptophan, and tyrosine are aromatic amino acids that are metabolized by hosts and microorganisms to produce compounds such as indoles and phenols, which are associated with various diseases, such as gastrointestinal diseases, metabolic diseases, and central nervous system diseases [47]. Phenylalanine is the precursor of catecholamine biosynthesis, and most of it is oxidized to tyrosine by phenylalanine hydroxylase in the body. The contents of phenylalanine and tyrosine in the body are considered closely related to insulin resistance [48]. Tryptophan is an essential amino acid for the synthesis of protein, which plays a key role in the microbiota-gut-brain axis [49]. Many bioactive compounds produced by tryptophan metabolism can regulate many physiological functions, including inflammation, metabolism, the immune response, and neurological function [50]. It could be found that the relative contents of L-tyrosine, L-tryptophan, and L-phenylalanine in 3-year-old PWR were significantly higher than those in PRR, suggesting that the metabolic pathway was more active and the accumulation of pharmacodynamic components was more abundant when the content was higher.

Similarly, KEGG analysis of the differential metabolites in the PWR showed that the differential metabolites of PW-1, PW-2, and PW-3 were mainly enriched in 76 metabolic pathways. The metabolic pathways with significant enrichment mainly included metabolic pathways, nicotinate and nicotinamide metabolism, glycerophospholipid metabolism, and galactose metabolism (Figure 9A). There were five differential compounds involved in the synthetic metabolic pathway of glycerophospholipid metabolism, of which three accumulated to a higher extent in PW-3, namely, choline, O-phosphorylethanolamine, and choline alfoscerate. There were eleven differential compounds involved in the metabolic pathway of galactose metabolism, and eight of them accumulated more in PW-3, including D-glucose, dulcitol, D-sorbitol, galactinol, and so on. There were eight differential compounds involved in the synthetic metabolic pathway of nicotinate and nicotinamide metabolism, and seven of them accumulated more in PW-3, which were succinic acid*, nicotinamide, L-aspartic acid, trigonelline, and so on. By analyzing all the differential metabolites in the significantly enriched pathway in 1~3-year-old PWR, it arose that the content was higher, mainly in the PW-3 stage.

KEGG analysis of differential metabolites in PRR showed that the differential metabolites of PR-1, PR-2, and PR-3 were mainly enriched in 83 metabolic pathways (Figure 8B). The metabolic pathways that were significantly enriched mainly included tryptophan metabolism, tyrosine metabolism, D-amino acid metabolism, glycerophospholipid metabolism, and glycine, serine, and threonine metabolism. There were seven different compounds involved in the metabolic pathway of tryptophan metabolism, of which five compounds accumulated more in PR-3, such as indole, L-tryptophan, 5-hydroxytryptophan, etc. There were six differential compounds involved in the tyrosine metabolism pathway, of which three accumulated more in PR-3, namely, fumaric acid, succinic acid*, and P-coumaric acid. There were seventeen differential compounds involved in the D-amino acid metabolism pathway, of which nine accumulated at higher extent in PR-2, including putrescine, cadaverine, L-serine, L-threonine*, and so on. There were five differential compounds involved in the metabolic pathway of glycerophospholipid metabolism, three of which were more accumulated in PR-1, namely choline, L-serine, and diethanolamine. There were eight differential compounds involved in the metabolic pathway of glycine, serine, and threonine metabolism. Among them, three metabolites accumulated more in PR-1, which were choline, L-serine, and diethanolamine. By analyzing all the differential metabolites in the significantly enriched pathway of 1~3-year-old PRR, it could be found that the content was the highest mainly in PR-2 or PR-3.

Glycerophospholipid metabolism is one of the key metabolic pathways involved in myocardial ischemia. Glycerophospholipids are important components of biofilms, such as cell membranes. They function in predicting and identifying cardiovascular diseases [51,52] and play important roles in plant growth and development and the stress response. For example, alfalfa improves drought resistance by increasing the content of glycerophospholipids [53]. It was found that with the increase in growth years, the contents of 2-γ-linolenoyl-glycerol*, 1-α-linolenoyl-glycerol*, and 2-linoleoylglycerol* in PWR and PRR increased gradually, and their accumulation played an important role in the plant response and adaptation to abiotic stress, which was helpful to reduce the damage to plants caused by adversity. It was speculated that *P. kingianum* could increase plant resistance by improving the level of glycerophospholipid metabolism, maintaining the normal function of the cell membrane, promoting signal transduction in vivo, etc., which need further verification in follow-up research. In summary, it was speculated that the above three metabolic pathways and their metabolites could be used as some of the pharmacodynamic material bases for the treatment of related diseases, such as elevated blood glucose levels and blood pressure caused by lipid metabolism disorders, in addition to sugars and saponins in *P. kingianum*.

### 2.7. Expression Analysis of Differential Metabolites

By performing a metabonomic analysis of PWR and PRR in different growth years, we found that the levels of amino acids and derivatives and flavonoids were higher in different *P. kingianum* germplasms in the same year (Figure 7A–C), and the levels of amino acids and derivatives and lipids were higher in the same *P. kingianum* germplasm from different growth years (Figure 8A,B).

The results showed that when the growth year was one, amino acids and derivatives were distributed in both PWR and PRR. Among them, the levels of L-tyrosine, L-tryptophan, 5-hydroxytryptophan, and other compounds were higher in PW-1, and the levels of L-pyroglutamic acid, L-ornithine, L-aspartic acid, and other compounds were higher in PR-1 (Figure S2A). At 2~3 years old, the relative contents of most amino acids and derivatives (such as L-pyroglutamic acid, aspartic acid di-O-glucoside, L-leucine*, etc.) in PWR were higher than those in PRR, while the expression levels of amino acids and derivatives such as cyclo(Tyr-Ala), S-(methyl)glutathione, and cyclo(Ser-Pro) in PRR were higher (Figure S2B,C). It is worth noting that the contents of most flavonoids in 1~3-year-old PRR maintained relatively high levels, such as quercetin-3-O-rutinoside (rutin)*, narcissin*, chrysoeriol-6,8-di-C-glucoside, and other compounds, along with a few flavonoids such as tricin-O-saccharic acid and apigenin-6. The contents of tricin-O-saccharic acid, apigenin-6,8-C-diarabinoside*, apigenin-6-C-xyloside-8-C-β-D-arabinoside*, and apigenin-6-C-arabinosyl-8-C-xyloside* were higher in 1~3-year-old PWR.

Similarly, among 212 differential metabolites of 1~3-year-old PWR, the contents of most amino acids and derivatives (such as 5-hydroxy-L-tryptophan, L-aspartyl-L-phenylalanine, L-proline, etc.) and lipids such as choline alfoscerate, 2-linoleoylglycerol*, and 2-γ-linolenoyl-glycerol* increased significantly with the increase in PWR growth years (Figure S3A). Among them, the expression levels of lipids such as LysoPE 18:2(2n isomer) *, LysoPE 20:2*, and amino acids and derivatives such as L-tyramine and N-acetylthreonine were the highest in the PW-2 and the lowest in the PW-1. Among the 215 differential metabolites of 1~3-year-old PRR, the contents of most lipids were higher in PR-3 (Figure S3B), such as LysoPE 18:3*, linoleic acid, 13-HPODE, and other substances. With the increase in PRR growth years, the contents of most amino acids and derivatives, such as L-lysine, L-threonine*, L-glutamic acid*, and other compounds, decreased significantly. Among them, the expression levels of lipids such as γ-linolenic acid*, and α-linolenic acid*, and amino acids and derivatives such as L-methionine, tyramine, and homoarginine were the highest in PR-2 and the lowest in PR-1.

### 2.8. Analysis of the Ideal Harvest Time of Different Germplasms of P. kingianum

The contents and compositions of secondary metabolites in medicinal plants determine the optimal harvest time and the quality of the medicinal plant. It was reported that the tubers of *P. kingianum* growing for two years were used for asexual reproduction, and the third year after transplanting was used for excavation [54]. The medicinal materials were large, the biological yield was high, and the comprehensive economic output benefit was good. It was the best harvest time. Su et al. [55] found that the best harvest time of *P. kingianum* among different years was five years, and the number of tubers and fresh weight reached the maximum under the field planting and understory planting modes. Zhang et al. [20] used ATR-FTIR and UV-Vis spectroscopy to determine the polysaccharide content of *P. kingianum* from different growth years. It was found that the polysaccharide content of *P. kingianum* from different growth years was quite different. The best harvest time was the fourth year after planting, and the accumulation of polysaccharides could be promoted by inhibiting the development of fibrous roots. Currently, the commonly used reproduction methods of *P. kingianum* are mainly seed reproduction and rhizome reproduction. However, different seedling breeding methods might lead to different harvesting seasons of *P. kingianum*. Therefore, the harvest time of medicinal plants should be considered in combination with the accumulation of active ingredients and the growth stage of plants. There was evidence that with the extension of years, the available nutrients in the sections of *P. kingianum* were transferred to the new part, decreasing the dry weight. In addition, the longer the growth time, the tendency of aging and serious wilting manifested in the older sections of the local root, with a loose tissue structure and the loss of many active ingredients [21,56]. This showed that in the case of large-scale planting of perennial plants, the reasonable planning of their planting and harvesting cycle need to be comprehensively evaluated according to factors such as pest control, biological yield, and field management costs to maximize the comprehensive economic benefits as much as possible.

In this study, seed propagation was used to breed seedlings. By observing the cultivation and growth of two germplasms of *P. kingianum*, we found that the average ground diameter of plants from large to small was 3 years old > 2 years old > 1 year old, and the number of plants per cluster was 3 years old > 2 years old > 1 year old. In addition, our study also found that three-year-old PWR and PRR might show higher stress resistance than other growth years. It could be seen that the accumulation of the most effective components in PWR and PRR was mainly concentrated in the growth process of two ~ three years.

So far, there have been few studies on the suitable harvest time of *P. kingianum*, comparisons with the optimal harvest time of *P. kingianum*, as well as analyses of the influencing factors, such as the price of *P. kingianum* and the capital and manpower input in the construction of the base. Therefore, we believed that as the growth years increase, the active ingredients also accumulate before harvesting PWR and PRR, which also coincided with the current common choice of the harvesting year of the fruit ripening. This study provided an experimental basis for the determination of the reasonable harvest period of the two germplasms of *P. kingianum*. The relationship between the different harvest years of *P. kingianum* and the efficacy remains to be further verified in the future to determine the best harvest period of *P. kingianum* with more sufficient evidence.

## 3. Materials and Methods

### 3.1. Plant Materials and Treatment

In this study, seeds were used to propagate seedlings of *P. kingianum* and *P. kingianum* ‘Linyun 1′, and after 1 year of seedling rearing, the seedlings of the two germplasms were transplanted from 2018 to 2021 (4a, 3a, 2a, and 1a). At the beginning of March each year, the seedlings of the two germplasms were transplanted to the base of Linyun Biotechnology Development Co., Ltd., Dali City, Yunnan Province, China (25°9′5″ N, 100° 29′ 5″ E, 2374 m). This place was red loam with a deep soil layer, fertile and loose soil, good drainage, and rich humus. The rhizomes of the plant were derived from one-year-old *P. kingianum* (PR-1) and *P. kingianum* ‘Linyun 1′ (PW-1), two-year-old *P. kingianum* (PR-2) and *P. kingianum* ‘Linyun 1′ (PW-2), and three-year-old *P. kingianum* (PR-3) and *P. kingianum* ‘Linyun 1′ (PW-3). The phenotypic characteristics of the plants are shown in Figure 1. The rhizomes of different varieties and different growth years of *P. kingianum* were cut into about 3 mm thick slices and put into 5 mL frozen tubes in three replicates, then quickly placed in liquid nitrogen, and transferred to an −80 °C ultra-low temperature refrigerator for storage. These samples were subjected to a metabolic analysis at Wuhan Metvel Biotechnology Co., Ltd., Wuhan, Hubei Province, China.

### 3.2. Widely Targeted Metabolomic Method Based on UPLC-MS/MS

The freeze-dried rhizomes of *P. kingianum* were ground with a mixer mill (MM 400, Retsch, Newtown, PA, USA) and zirconia beads at 30 Hz for 1.5 min. A total of 100 mg of the powder was weighed and dissolved in 0.6 mL of 70% methanol for extraction; the dissolved sample was refrigerated at 4 °C overnight, during which time it was vortexed six times to improve the extraction rate. After centrifugation (speed 10,000× *g*, 10 min), the supernatant was taken, and the sample was filtered with a microporous membrane (0.22 μm pore size) and stored in an injection bottle for the UPLC-MS/MS analysis.

UPLC-MS/MS systems (including a Shim-pack UFLC Shimadzu CBM30A system and Applied Biosystems 4500 QTRAP (Thermo Fisher Scientific (Waltham, MA, USA)) were used to analyze the rhizome extract of *P. kingianum*. The chromatographic column was the Agilent SB-C18 chromatographic column (Agilent Technologies (Santa Clara, CA, USA) (1.8 μm, 2.1 mm, 100 mm), with water (0.1% formic acid) and acetonitrile as the mobile phase. The elution gradient was 5:95 *v*/*v* at 0 min, 95:5 *v*/*v* at 9.0 min, 95:5 *v*/*v* at 10.0 min, 5:95 *v*/*v* at 11.0 min, and 5:95 *v*/*v* at 14.0 min; the flow rate was 0.35 mL/min; the temperature was 40 °C; and the injection volume was 4 μL.

LIT and triple quadrupole (QQQ) scans were performed in a triple quadrupole linear ion trap mass spectrometer (Q TRAP) and an AB4500 Q TRAP UPLC/MS/MS system (AB Sciex, Framingham, MA, USA). The system was equipped with an ESI turbo ion spray interface and used positive and negative ion modes for data acquisition. The operating parameters of the ESI source were as follows: the source temperature was 550 °C; the ion spray voltage was 5500 V; the ion source gas I, gas II, and curtain gas (CUR) were set to 50, 60 and 25.0 psi, respectively; and the collision-activated dissociation (CAD) was set to high. In QQQ and LIT modes, the instrument was tuned and calibrated with 10 and 100 μmol/L polypropylene glycol aqueous solutions, respectively. The triple quadrupole was scanned using multi-reaction detection mode (MRM), and the collision gas (nitrogen) was set to medium. In QQQ, each ion pair was scanned and detected according to the optimized declustering potential (DP) and collision energy (CE).

### 3.3. Metabolite Data Processing and Statistical Analysis

Before data analysis, a quality control (QC) analysis was carried out to confirm the reliability of the data. The extracts of three samples were mixed to make quality control samples, and one quality control sample was inserted into every three samples to monitor the changes in the repeated analysis. The qualitative analysis of metabolites was based on MWDB V 2.0 (Matville Biotechnology Co., Ltd., Wuhan, Hubei Province, China) and secondary spectrum information, and the quantitative analysis was based on MRM of triple quadrupole mass spectrometry. Principal component analysis (PCA) and orthogonal partial least squares discriminant analysis (OPLS-DA) were used to analyze the two groups of samples. According to the variable important projection value (VIP) ≥ 1 of the OPLS-DA models, combined with a *p*-value < 0, and fold change (FC) ≥ 2 or FC ≤ 0.5, the differential metabolites were screened. The original relative contents of the differential metabolites obtained by screening and identification were normalized by rows, and the heat map was drawn using the Complex Heatmap package of R software, version 4.0.2. At the same time, the differential metabolites were submitted to the Kyoto Encyclopedia of Genes and Genomes (KEGG) database (http://www.kegg.jp/kegg/pathway.html, accessed on 23 May 2024) for the related pathway enrichment analysis.

## 4. Conclusions

In this study, UPLC-MS/MS and multivariate statistical analysis were used for the first time to study the metabolic characteristics of *P. kingianum* rhizomes from different varieties and different growth stages. The results showed that a total of 418 metabolites were identified in two varieties and three growth years. The total amounts and types of metabolites detected in PWR were higher than those in PRR. The screening of differential metabolites showed that the contents of flavonoids and organic acids were richer in PRR, and the contents of lipids, phenolic acids, and amino acids and derivatives were higher in PWR. It was noteworthy that most of the differential compounds were mainly enriched in PRR when the growth year was one. In the third year, PWR had a greater variety and higher contents of differential compounds, and the expression of several metabolites was significantly higher than that in PRR, i.e., the relative content was increased, which indicated that PWR accumulated more metabolites relative to PRR. In addition, we also found that the relative contents of flavonoids, phenolic acids, and alkaloids were the highest in PW-1. PW-3 had high levels of amino acids and derivatives, organic acid, saccharides and alcohols, and lipids. The relative contents of amino acids and derivatives, flavonoids, phenolic acids, and alkaloids were the highest in PR-1, and PR-3 had high levels of organic acid, saccharides and alcohols, and lipids. Our analysis of KEGG metabolic pathways in different germplasms of *P. kingianum* rhizome from the same growth year revealed that significantly enriched metabolic pathways (tryptophan metabolism; biosynthesis of amino acids; phenylalanine, tyrosine, and tryptophan biosynthesis; and plant hormone signal transduction) were more actively biosynthesized in PWR, and their metabolites were expressed at higher levels in the third year. In the same germplasm of *P. kingianum* rhizome from different years, the metabolites involved in PRR were mainly the highest in PR-2 and PR-3, and the metabolites involved in PWR were mainly the highest in PW-3. The above results indicate that with the increase in growth years, the expression of metabolites in both types of *P. kingianum* increases, and the types and quantities of metabolites in PWR are more than those in PRR. The research results provide theoretical and practical references for cultivating new varieties of *P. kingianum* with excellent quality, yield, and stress resistance. In addition, these findings can be used in genomics and transcriptomics to screen target genes that regulate the ideal shape of *P. kingianum*.

## Figures and Tables

**Table 1:** Investigation of the growth of plants in the cultivation test (PW vs. PR).

Parameter	One Year Old		Two Years Old		Three Years Old		Four Years Old	
	PW	PR	PW	PR	PW	PR	PW	PR
Average plant height (m)	0.35	0.36	0.69	0.79	1.78	2.21	1.92	2.63
Increase (%)	−0.03		−0.13		−0.19		−0.27	
Average ground diameter (cm)	0.28	0.27	0.35	0.32	0.73	0.71	0.81	0.71
Increase (%)	0.04		0.09		0.03		0.14	
Plant number (plant/clump)	1.5	1.3	3	2.6	15.8	10.02	18.8	14.02
Increase (%)	0.15		0.15		0.58		0.34	

**Table 2:** Survey of tuber yield in the cultivation experiment (PW vs. PR).

Parameter	Four Years Old	
	PW	PR
Average individual yield (kg)	0.88	0.51
Increase (%)	72.55	
Average yield per mu (kg)	6918.7	3616.5
Increase (%)	91.31	

**Table 3:** Comparison of differences in chemical constituents contained in PWR and PRR from different growth years.

Category	PW-1 vs. PR-1				PW-2 vs. PR-2				PW-3 vs. PR-3			
	TNM ^1^	TNDM ^2^	NUR ^3^	NDR ^4^	TNM	TNDM	NUR	NDR	TNM	TNDM	NUR	NDR
Lipids	68	24	11	13	68	22	12	10	68	23	9	14
Amino acids and derivatives	67	21	12	9	67	25	9	16	67	31	4	27
Alkaloids	42	18	9	9	42	18	12	6	39	17	5	12
Phenolic acids	42	23	17	6	42	16	6	10	42	17	5	12
Flavones	41	27	23	4	41	28	24	4	41	22	17	5
Organic acids	38	9	6	3	38	12	6	6	38	13	7	6
Nucleotides and derivatives	31	11	3	8	31	14	4	10	31	10	2	8
Lignans and Coumarins	15	10	6	4	15	10	5	5	15	7	4	3
Terpenoids	3	1	1	0	3	0	0	0	3	0	0	0
Steroids	1	1	0	1	1	1	0	1	1	1	0	1
Others	70	30	26	4	70	24	8	16	69	12	8	4

^1^ TNM: total number of metabolites; ^2^ TNDM: total number of differential metabolites; ^3^ NUR: number of up-regulated; ^4^ NDR: number of down-regulated.

## Data Availability

Data from this study are included in this article/Supplementary Materials; further inquiries can be directed to the corresponding author.

## Supplementary Materials

The following supporting information can be downloaded at https://www.mdpi.com/article/10.3390/molecules29215180/s1: Figure S1. Screening results for shared differential metabolites in PWR and PRR in different growth years. Figure S2. Relative amounts of the highest differential metabolites in different germplasms of *P. kingianum*. Figure S3. Relative amounts of the highest differential metabolites in *P. kingianum* from different growth years. Table S1. Metabolites found in different germplasms and different growth years of *P. kingianum.* Table S2. OPLS-DA model validation values of PWR and PRR in different growth years. Table S3. Shared differential metabolites in 1~3-year-old PWR (PW-1 vs. PW-2 vs. PW-3). Table S4. Shared differential metabolites in 1~3-year-old PRR (PR-1 vs. PR-2 vs. PR-3).

## References

[1] Jin J., Lao J., Zhou R., He W., Qin Y., Zhong C., Xie J., Liu H., Wan D., Zhang S. (2018). Simultaneous identification and dynamic analysis of saccharides during steam processing of rhizomes of *Polygonatum cyrtonema* by HPLC–QTOF–MS/MS. Molecules.

[2] Chinese Pharmacopoeia Commission (2020). Pharmacopoeia of the People’s Republic of China: Part 1.

[3] Li R., Tao A., Yang R., Fan M., Zhang X., Du Z., Shang F., Xia C., Duan B. (2020). Structural characterization, hypoglycemic effects and antidiabetic mechanism of a novel polysaccharides from *Polygonatum kingianum* coll. et hemsl. Biomed. Pharmacother..

[4] Gu W., Wang Y., Zeng L., Dong J., Bi Q., Yang X., Che Y., He S., Yu J. (2020). Polysaccharides from *Polygonatum kingianum* improve glucose and lipid metabolism in rats fed a high fat diet. Biomed. Pharmacother..

[5] Yelithao K., Surayot U., Lee J.H., You S. (2016). RAW264. 7 cell activating glucomannans extracted from rhizome of *Polygonatum sibiricum*. Prev. Nutr. Food Sci..

[6] Yang J.J., Zhang X., Dai J.F., Ma Y.G., Jiang J.G. (2023). Effect of fermentation modification on the physicochemical characteristics and anti-aging related activities of *Polygonatum kingianum* polysaccharides. Int. J. Biol. Macromol..

[7] Qin P.Y., Xu Y.J., Zuo X.D., Duan J.H., Qiu B., Li X.F., Li J.P., Yu J. (2022). Effect and mechanisms of *Polygonatum kingianum* (polygonati rhizome) on wound healing in diabetic rats. J. Ethnopharmacol..

[8] Huyen D.T.T., Phuong N.T., Hien N.T.T., Nga N.T., Hung L.N., Thao D.T., Ha L.M. (2020). *Polygonatum kingianum* rhizome extract alleviates collagen antibody-induced arthritis by modulating proinflammatory cytokine production in mice. Asian Pac. J. Trop. Biomed..

[9] Yin S., Qian L. (2022). Research progress in diversity of origin plants of Chinese medicinal *Polygonati* rhizoma and its utilization. Chin. Wild Plant Resour..

[10] Yang L. (2023). The development status and countermeasures of *Polygonatum* industry under the rural revitalization strategy. New Agric..

[11] Cheng Z., Li H., Wang Y., Zhou M., Wang C., Song Y., Ruan P., Zhang X. (2023). Development status and countermeasures of *Polygonatum* industry in Bijie city. Agric. Eng..

[12] Li Y., Kong D., Fu Y., Sussman M.R., Wu H. (2020). The effect of developmental and environmental factors on secondary metabolites in medicinal plants. Plant Physiol. Biochem..

[13] Ning K., Hou C., Wei X., Zhou Y., Zhang S., Chen Y., Yu H., Dong L., Chen S. (2022). Metabolomics analysis revealed the characteristic metabolites of hemp seeds varieties and metabolites responsible for antioxidant properties. Front. Plant Sci..

[14] Qu X., Hu S., Li T., Zhang J., Wang B., Liu C. (2022). Metabolomics analysis reveals the differences between *Bupleurum Chinense* DC. And *Bupleurum scorzonerifolium* Willd. Front. Plant Sci..

[15] Li Z., Wang H., Feng L., Song L., Lu Y., Li H., Li Y., Tian G., Yang Y., Li H. (2022). Comparative metabolomics provides novel insights into correlation between dominant habitat factors and constituents of *Stellaria radix* (*Stellaria dichotoma* L. var. *lanceolata* Bge.). Front. Plant Sci..

[16] Yuan Y., Zuo J., Zhang H., Li R., Yu M., Liu S. (2022). Integration of transcriptome and metabolome provides new insights to flavonoids Biosynthesis in *Dendrobium huoshanense*. Front. Plant Sci..

[17] Golubkina N.A., Kharchenko V.A., Moldovan A.I., Koshevarov A.A., Zamana S., Nadezhkin S., Soldatenko A., Sekara A., Tallarita A., Caruso G. (2020). Yield, growth, quality, biochemical characteristics and elemental composition of plant parts of celery leafy, stalk and root types grown in the Northern Hemisphere. Plants.

[18] Li A., Li W., Wu W., Zhang T., Zhang J., Wu Y., Liu R. (2023). Comparison of flavonoids, amino acids and phenolic acids in different *Polygonatum* rhizomes. Chin. J. Appl. Environ. Biol..

[19] Yang L., Wen K.S., Ruan X., Zhao Y.X., Wei F., Wang Q. (2018). Response of plant secondary metabolites to environmental factors. Molecules.

[20] Zhang J., Wang Y.Z., Yang M.Q., Yang W.Z., Yang S.B., Zhang J.Y. (2021). Identification and evaluation of *Polygonatum kingianum* with different growth ages based on data fusion strategy. Microchem. J..

[21] Zhang P., Wang J., Gong Y., Zhang X., Zhang Y. (2007). Dry matter accumulation and polysaccharides distribution in different sections of perennial *Polygonatum sibiricum* red. Acta Bot. Boreali-Occident. Sin..

[22] Yang M., Meng F., Gu W., Fu L., Zhang F., Li F., Tao Y., Zhang Z., Wang X., Yang X. (2021). Influence of polysaccharides from *Polygonatum kingianum* on short-chain fatty acid production and quorum sensing in *Lactobacillus faecis*. Front. Microbiol..

[23] He S., Wang X., Chen J., Li X., Gu W., Zhang F., Cao G., Yu J. (2022). Optimization of the ultrasonic-assisted extraction technology of steroidal saponins from *Polygonatum kingianum* collett & hemsl and evaluating its quality planted in different areas. Molecules.

[24] Segla K.D.S., Xu F., You J., Zhou R., Li D., Wang L. (2022). Widely targeted metabolome profiling of different colored sesame (*Sesamum indicum* L.) seeds provides new insight into their antioxidant activities. Food Res Int..

[25] Xue G., Su S., Yan P., Shang J., Wang J., Yan C., Li J., Wang Q., Xiong X., Xu H. (2022). Integrative analyses of widely targeted metabolomic profiling and derivatization-based LC-MS/MS reveals metabolic changes of Zingiberis Rhizoma and its processed products. Food Chem..

[26] Liang X., Wang Y., Li Y., An W., He X., Chen Y., Shi Z., He J., Wan R. (2022). Widely-Targeted metabolic profiling in *Lyciumbarbarum* fruits under salt-alkaline stress uncovers mechanism of salinity tolerance. Molecules.

[27] Chen L., Tian M., Jin B., Yin B., Chen T., Guo J., Tang J., Cui G., Huang L. (2022). Integrating metabolomics and transcriptomics to unveil atisine Biosynthesis in *Aconitum gymnandrum* maxim. Int. J. Mol. Sci..

[28] Darwish R.S., El-Banna A.A., Ghareeb D.A., El-Hosseny M.F., Seadawy M.G., Dawood H.M. (2022). Chemical profiling and unraveling of anti-COVID-19 biomarkers of red sage (*Lantana camara* L.) cultivars using UPLC-MS/MS coupled to chemometric analysis, in vitro study and molecular docking. J. Ethnopharmacol..

[29] Fan Y., Cao X., Zhang M., Wei S., Zhu Y., Ouyang H., He J. (2022). Quantitative comparison and chemical profile analysis of different medicinal parts of *Perilla frutescens* (L.) Britt. from different varieties and harvest periods. J. Agric. Food Chem..

[30] Xiao L., Li X., Wu T. (2022). A new *Polygonatum kingianum* cultivar ‘linyun 1’. Acta Acta Hortic. Sinic..

[31] Xie L., Xiao L., Wu T., Li X., Su B., Liu Z. (2022). Genetic relationship of *Polygonatum kingianum* with three different flower colors. J. Zhejiang For. Sci. Technol..

[32] Hou S., Men Y., Wei M., Zhang Y., Li H., Sun Z., Han Y. (2022). Total protein content, amino acid composition and eating-quality vealuation of foxtail millet (*Setaria italica* (L.) P. *Beauv*). Foods.

[33] Ling Z.N., Jiang Y.F., Ru J.N., Lu J.H., Ding B., Wu J. (2023). amino acid metabolism in health and disease. Signal Transduct. Target. Ther..

[34] Li H., An L. (2019). Research status of determination and analysis technology of amino acid content in Chinese medicinal materials. Rural. Econ. Sci. Technol..

[35] Zhai L., Wang X. (2018). Syringaresinol-di-O-β-D-glucoside, a phenolic compound from *Polygonatum sibiricum*, exhibits an antidiabetic and antioxidative effect on a streptozotocin-induced mouse model of diabetes. Mol. Med. Rep..

[36] Xing Z. (2016). Research progress on lipid metabonomics. Feed Rev..

[37] Lu Z., Jiang L. (2018). Analysis of current status and trends of patent application of glycerophosphoryl choline in medical area. China Invent. Pat..

[38] Calis Z., Mogulkoc R., Baltaci A.K. (2020). The roles of flavonoles/flavonoids in neurodegeneration and neuroinflammation. Mini-Rev. Med. Chem..

[39] Ding H., Wen D., Fu Z., Qian H. (2013). The secretion of organic acids is also regulated by factors other than aluminum. Environ. Monit. Assess..

[40] Zhang D., Nie S., Xie M., Hu J. (2020). Antioxidant and antibacterial capabilities of phenolic compounds and organic acids from *Camellia oleifera* cake. Food Sci. Biotechnol..

[41] Tao J., He S., Kong L., Cheng G. (2022). The protective effect of 6’-O-caffeoylarbutin on type l diabetes. J. Chin. Inst. Food Sci. Technol..

[42] Alasmary F.A., Awaad A.S., Alqahtani S.M., El-Meligy R.M., Abdullah D.A., Alqasoumi S.I. (2020). Evaluation of the chemical constituents and potential biological activities of *Cunninghamella blakesleeana*. Saudi Pharm..

[43] Khan M.F., Murphy C.D. (2021). 3-hydroxytyrosol regulates biofilm growth in *Cunninghamella elegans*. Fungal Biol..

[44] Hof C., Khan M.F., Murphy C.D. (2023). Endogenous production of 2-phenylethanol by *Cunninghamella echinulata* inhibits biofilm growth of the fungus. Fungal Biol..

[45] Singh R., Kumar M., Mittal A., Mehta P.K. (2017). Microbial metabolites in nutrition, healthcare and agriculture. 3 Biotech.

[46] Kuehl F.A., Egan R.W. (1980). Prostaglandins, arachidonic acid, and inflammation. Science.

[47] Bai X., Hu T., Xue X., Dong X. (2023). Metabolism of phenylalanine in host andintestinal microorganism: Research progress. Chin. J. Microecol..

[48] Ottosson F., Ericson U., Almgren P., Nilsson J., Magnusson M., Fernandez C., Melander O. (2016). Postprandial levels of branch chained and aromatic amino acids associate with fasting glycaemia. J. Amino Acids..

[49] Roth W., Zadeh K., Vekariya R., Ge Y., Mohamadzadeh M. (2021). Tryptophan metabolism and gut-brain homeostasis. Int. J. Mol. Sci..

[50] Xue C., Li G., Zheng Q., Gu X., Shi Q., Su Y., Chu Q., Yuan X., Bao Z., Lu J. (2023). Tryptophan metabolism in health and disease. Cell Metab..

[51] Stegemann C., Pechlaner R., Willeit P., Langley S.R., Mangino M., Mayr U., Menni C., Moayyeri A., Santer P., Rungger G. (2014). lipidomics profiling and risk of cardiovascular disease in the prospective population-based Bruneck study. Circulation.

[52] Takeda H., Koike T., Izumi Y., Yamada T., Yoshida M., Shiomi M., Fukusaki E., Bamba T. (2015). lipidomic analysis of plasma lipoprotein fractions in myocardial infarction-prone rabbits. J. Biosci. Bioeng..

[53] Xu H., Li X. (2020). A metabolomics analysis of the effect of water deficit on the freezing tolerance of *Medicago sativa*. Acta Prat. Sinic..

[54] Lu D., Hu Y., Huang Z., Feng E., Nie Y., Wang C., Yuan L., Li H., Rao G. (2023). Experiments of agronomic traits and yield of *Polygonatum kingianum* with different growth years. Yunnan Agric. Sci. Technol..

[55] Su W., Zhao Y., Wu X., Xiong X., Su Y., Su Z. (2022). Effects of different planting patterns and years on yield and quality of *Polygonatum kingianum*. J. West. China For. Sci..

[56] Liu J., Zhu X., Ye H., Wang W., Ye Y., Hai M. (2017). The appropriate collection period of *Polygonatum cyrtonema* in Yunnan. Chin. Agric. Sci. Bull..

