# Supplementary material for: Metabolomic Diversity in Polygonatum kingianum Across Varieties and Growth Years
Source: Molecules. 2024 Nov 1;29(21):5180. doi: 10.3390/molecules29215180 (PMC11547710; doi:10.3390/molecules29215180)
Supplement: Supplementary file 1 [file molecules-29-05180-s001.zip › molecules-3222358-Supplementary Material.pdf]

Supplementary Material

Widely Targeted Metabolomics Reveals Metabolite Diversity in *Polygonatum kingianum* and its New varieties with Different Growth Years

1 Supplementary Figures and Tables

1.1 Supplementary Figures

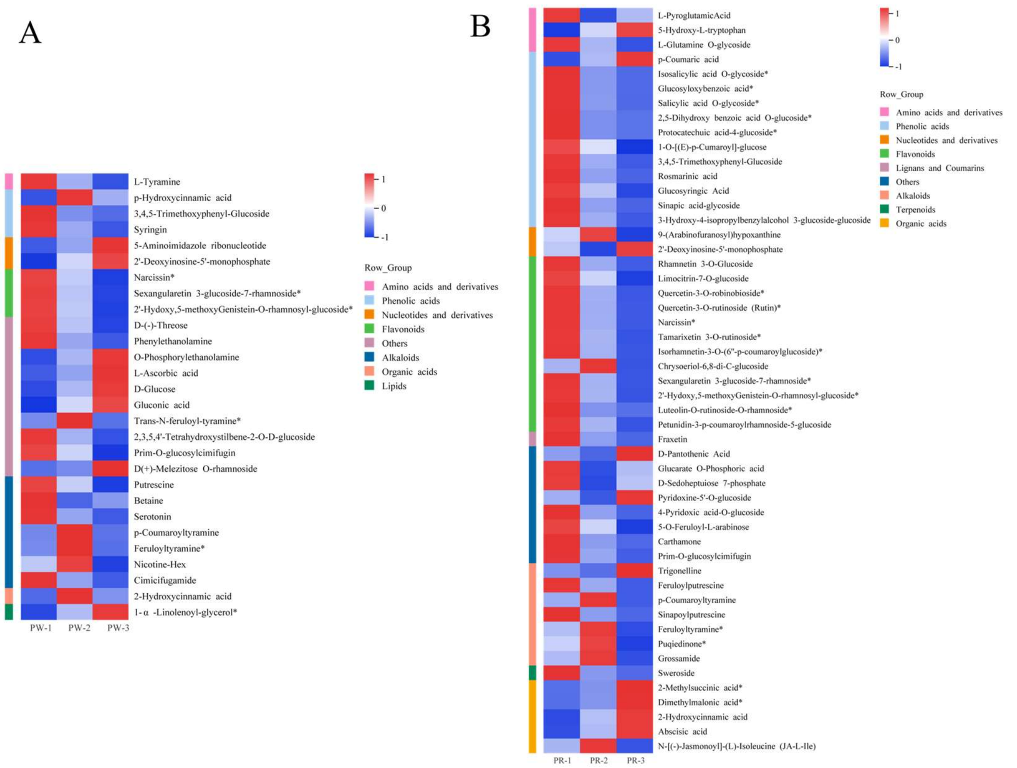

Figure S1. Screening results of shared differential metabolites in PWR and PRR at different growth years. (**A**) heat map of 28 shared differential metabolites in 1~3-year-old PWR, and (**B**) heat map of 51 shared differential metabolites in 1~3-year-old PRR.

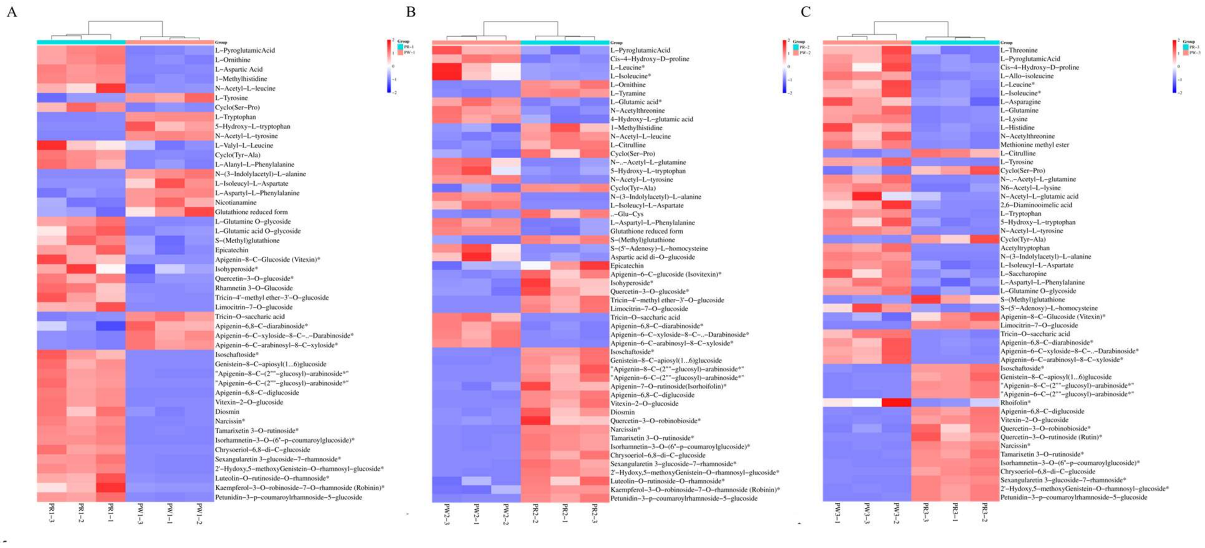

Figure S2. Relative amounts of the highest differential metabolites in different germplasms *P. kingianum*. (**A-C**) Heatmaps of amino acids and derivatives and flavonoids in PW-1 vs. PR-1, PW-2

vs. PR-2, and PW-3 vs. PR-3, with red and blue colors representing high and low expression, respectively.

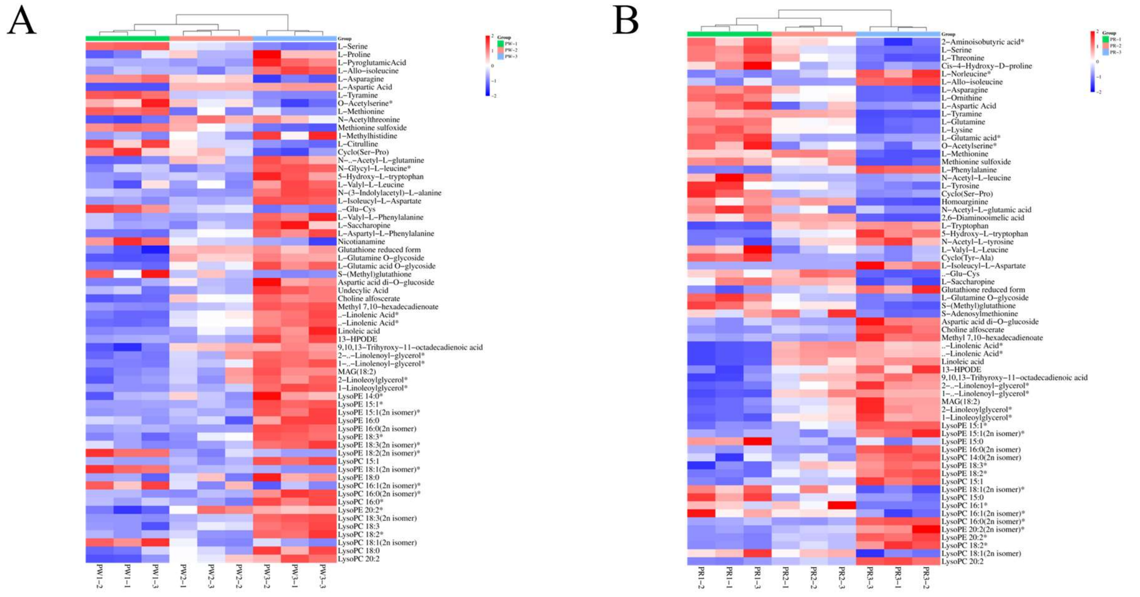

Figure S3. Relative amounts of the highest differential metabolites in different growth years *P. kingianum*. (**A-B**) Heatmaps of amino acids and derivatives and lipids in PW-1 VS. PW-2 VS. PW-3 and PR-1 VS. PR-2 VS. PR-3, with red and blue colors representing high and low expression, respectively.

1.2 Supplementary Tables

Table S1. Metabolites found in different germplasms and different growth years of *P. kingianum.*

Table S2. OPLS-DA model validation values of PWR and PRR in different growth years.

Table S3. Shared differential metabolites in 1~3-year-old PWR (PW-1 VS. PW-2 VS. PW-3).

Table S4. Shared differential metabolites in 1~3-year-old PRR (PR-1 VS. PR-2 VS. PR-3).
